# Differentially methylated CpGs in response to growth hormone administration in children with idiopathic short stature

**DOI:** 10.1186/s13148-022-01281-z

**Published:** 2022-05-18

**Authors:** Xiaojian Shao, Catherine Le Stunff, Warren Cheung, Tony Kwan, Mark Lathrop, Tomi Pastinen, Pierre Bougnères

**Affiliations:** 1 Digital Technologies Research Center, National Research Council Canada, Ottawa, ON K1A 0R6 Canada; 2 UMR INSERM 1195 and Université Paris Saclay, Endocrinologie Pédiatrique, Hôpital Bicêtre, 94276 Le Kremlin-Bicêtre Cedex, France; 3 Genomic Medicine Center, Children’s Mercy - Kansas City and Children’s Mercy Research Institute, Kansas City, MO 64108 USA; 4 Department of Human Genetics, McGill University and McGill Genome Center, Montreal, QC H3A 0G1 Canada

**Keywords:** Pharmacoepigenomics, Growth hormone, DNA methylation, Intervention epigenetics, Idiopathic short stature

## Abstract

**Background:**

Recombinant human growth hormone (rhGH) has shown a great growth-promoting potential in children with idiopathic short stature (ISS). However, the response to rhGH differs across individuals, largely due to genetic and epigenetic heterogeneity. Since epigenetic marks on the methylome can be dynamically influenced by GH, we performed a comprehensive pharmacoepigenomics analysis of DNA methylation changes associated with long-term rhGH administration in children with ISS.

**Results:**

We measured DNA methylation profiles before and after GH treatment (with a duration of ~ 18 months in average) on 47 healthy children using customized methylC-seq capture sequencing. Their changes were compared and associated with changes in plasma IGF1 by adjusting sex, age, treatment duration and estimated blood proportions. We observed a considerable inter-individual heterogeneity of DNA methylation changes responding to GH treatment. We identified 267 response-associated differentially methylated cytosines (DMCs) that were enriched in promoter regions, CpG islands and blood cell-type-specific regulatory elements. Furthermore, the genes associated with these DMCs were enriched in the biology process of “cell development,” “neuron differentiation” and “developmental growth,” and in the *TGF*-beta signaling pathway, *PPAR* Alpha pathway, endoderm differentiation pathway, adipocytokine signaling pathway as well as PI3K-Akt signaling pathway, and cAMP signaling pathway.

**Conclusion:**

Our study provides a first insight in DNA methylation changes associated with rhGH administration, which may help understand mechanisms of epigenetic regulation on GH-responsive genes.

**Supplementary Information:**

The online version contains supplementary material available at 10.1186/s13148-022-01281-z.

## Introduction

Growth hormone (GH) acts directly on intracellular pathways downstream of the GH receptor or via the stimulation and action of IGF1 [1]. Physiological effects of GH are mainly on growth, body composition and metabolism. In the epiphyseal growth plate, GH effects are largely mediated by promoting IGF1 production to stimulate skeletal growth. In addition, GH is known to increase muscle mass. GH also contributes to the acute metabolic response to stressful situations, such as fasting [2, 3], exercise [4], injuries, critical illnesses [5] and infectious diseases [6] by acting on the liver, pancreatic beta cells, adipose tissue and muscle. Overall, most of these GH effects are mediated by activating the transcription of numerous genes.

Non-physiological exposure to GH occurs in the human clinic when children with short stature (SS) receive supra-physiological doses of recombinant human GH (rhGH) to stimulate their skeletal growth. These cases differ widely in the level and duration of exposure to rhGH which may leave lasting marks in different tissues that could conceivably be based on epigenetic mechanisms.

Changes in CpG methylation of the genome are one of the most studied epigenetic mechanisms. They can facilitate or reduce the transcription of certain genes when they occur in the regulatory zones of these genes. Once installed, some of them cannot be erased. However, they can show a certain plasticity and vary with age or environmental factors [7–11]. Most of them are variable depending on the tissue [12–14]. Modifications of CpG methylation have been associated with cancer, metabolic and cardiovascular diseases [15–17]. Following binding to its receptor, GH activates GHR-associated Src family kinases, acting via other intracellular pathways [18], such as ERK and Jun*,* which are known to affect CpG methylation [19].

It was therefore interesting to investigate whether GH was capable of producing its own epigenetic marks on the methylome. The only opportunity to detect them in the human clinic is to study the methylome before and after exposure to the hormone. This is made possible in one particular circumstance, the application of rhGH treatment to normal small children. Given the considerable individual variability of methylation marks, it would have been difficult to compare rhGH treated with untreated children. Therefore, we searched for acquired CpG modifications by studying the same children before and during rhGH exposure. As for most DNA methylation studies in humans, it was only possible to study blood cells in these children, with the expectation that changes in blood cells may reflect those occurring in the physiological target tissues of GH action. It is probably important to clarify that our study does not address at all the epigenetic variations that could contribute to the mechanisms of short stature, which have been the subject of a number of previous studies. Such methylation marks are not expected to vary upon GH administration and are therefore outside the scope of our approach devoted to the epigenetic consequences of exposure to GH action.

## Methods

### Cohort—sample collection

DNA samples were obtained from 47 children with non-pathological idiopathic short stature (ISS) recruited among 317 children who served as controls in a study of type 1 diabetes (T1D) epigenetics [20]. The 47 studied children with short stature all had ISS, as defined [21]. Briefly, this diagnosis requires that child’s height is < − 2SD (standard deviation) from the population average, with a birth length > − 2SD for gestational age (none of them had intrauterine growth retardation) and appropriate for parents’ height, then linear and normal growth rate, and no detectable etiology for the short stature, such as chromosomal, endocrine, or skeletal diseases. Classically, testing of such short children includes clinical examination, bone X-rays, IGF1measurement and/or GH stimulation tests (to exclude GH deficiency). The 47 studied children all went through these screening analyses, and none of them showed any abnormality in the cited parameters. This study was supported by the Programme Hospitalier de Recherche Clinique of the French Ministry of Health according to the French bioethics law with the objective of studying gene–environment factors in young patients with T1D and age-matched controls. Families were carefully informed about the investigational nature of the study and signed an informed consent agreed by CPP (number DC-2008-693; NI 2620, Comité de Protection des Personnes). The studied children had received rhGH treatment for a duration of 6–38.4 months.

None had deficiencies of GH or other hormones, chromosomal disorders or syndromes, skeletal dysplasia or metabolic disease. All children received rhGH as daily sc injections 6 days/wk, starting with a 40 µg/kg day rhGH dose and following a target-to-treat rhGH dosing protocol based on the growth response to treatment, so that individual average dose ranged 40–113 µg/kg day across the studied children. Children gave two blood samples, one before onset of rhGH treatment and the other after 6–38.4 months of rhGH treatment. All were seen as outpatients every 6 months for clinical examination and measurements of serum IGF-I. Children were healthy at time of study, with no sign of viral or other intercurrent infection. Blood samples were collected in the course of routine medical evaluation of patients. DNA methylation was measured in peripheral blood mononuclear cells (PBMC) samples from these patients before and during treatment.

### Isolation of genomic DNA

Peripheral blood mononuclear cells (PBMC) were isolated from fresh blood using a density gradient. Five milliliters of fresh blood were mixed with 5 ml of NaCl 154 mM, and 5 ml of Lymphoprep solution (Eurobio, Paris, France) was added to the diluted blood and centrifuged for 20 min at room temperature at 800 g. After centrifugation, the interphase containing PBMC was carefully aspirated and the cells were mixed with NaCl. The cell suspension was centrifuged at 300 g and the cell pellet washed with PBS. PBMC were frozen at − 80 °C. Nucleic acids were extracted from PBMC using Gentra Puregene blood kit (Qiagen, Hilden, Germany).

### Measurement of IGF1 concentrations

IGF1 concentrations were measured in serum samples around 07.00 am to 08.00 pm before breakfast using a chemiluminescent immunometric assay after pre-treatment with acid using Immulite®2000 (Siemens Healthcare Diagnostics Products Llanberis, UK). IGF1 values under treatment were averaged for analysis.

### DNA methylation capture sequencing

Methylation capture sequencing (MCC-Seq) was performed as previously described [22–24]. In brief, the MCC-Seq protocol was carried out using the SeqCap Epi Enrichment System protocol (Roche NimbleGen) [22–24]. Specifically, a whole-genome sequencing library was prepared and bisulfite converted, amplified and then a capture enriching for targeted bisulfite-converted DNA fragments was carried out. Equal amounts of multiplexed libraries (12 samples per capture) were combined and were further amplified. Lastly, the MCC-Seq libraries were sequenced on the Illumina HiSeq4000 or NovaSeq 6000 system using 100 bp paired-end sequencing. More specifically, whole-genome sequencing libraries were generated from 700 to 1000 ng of genomic DNA spiked with 0.1% (w/w) unmethylated λ DNA (Promega) previously fragmented to 300–400 bp peak sizes using the Covaris focused-ultrasonicator E210. Fragment size was controlled on a Bioanalyzer DNA 1000 Chip (Agilent), and the KAPA High Throughput Library Preparation Kit (KAPA Biosystems) was applied. End repair of the generated dsDNA with 3′- or 5′-overhangs, adenylation of 3′-ends, adaptor ligation and clean-up steps were carried out as per KAPA Biosystems' recommendations. The cleaned-up ligation product was then analyzed on a Bioanalyzer High Sensitivity DNA Chip (Agilent) and quantified by PicoGreen (Life Technologies). Samples were then bisulfite converted using the Epitect Fast DNA Bisulfite Kit (Qiagen), according to the manufacturer's protocol. Bisulfite-converted DNA was quantified using OliGreen (Life Technologies) and, based on quantity, amplified by 9–12 cycles of PCR using the Kapa Hifi Uracil + DNA polymerase (KAPA Biosystems), according to the manufacturer's protocol. The amplified libraries were purified using Ampure Beads and validated on Bioanalyzer High Sensitivity DNA Chips, and quantified by PicoGreen.

The hybridization procedure of the amplified bisulfite-converted library was performed as described by the manufacturer, using 1 μg of total input of library, which was evenly divided by the libraries to be multiplexed, and incubated at 47 °C for 72 h. Washing and recovering of the captured library, as well as PCR amplification and final purification, were carried out as recommended by the manufacturer. Quality, concentration and size distribution of the captured library were determined by Bioanalyzer High Sensitivity DNA Chips.

This blood MCC-Seq panel covers (1) the majority of human gene promoters, blood-cell-lineage-specific enhancer regions and methylation footprint regions [25] observed in blood, (2) CpGs from Illumina Human Methylation 450 Bead Chips and (3) published autoimmune-related SNPs as well as SNPs in their LD regions with r2 > 0.8. Overall, it covers 4,861,805 CpGs which have been applied to multiple blood-based epigenome-wide association studies [24, 26].

### MCC-Seq data process

Targeted MCC-Seq HiSeq and NovaSeq reads were aligned using the Epigenome Pipeline available from the DRAGEN Bio-IT platform (Edico Genomics/Illumina). Specifically, the MCC-Seq paired-end raw reads were first demultiplexed into FASTQ files using Illumina’s bcl2Fastq2-2.19.1 software. Reads were then trimmed for quality (phred33 ≥ 20) and Illumina adapters using trimgalore v.0.4.2 (https://www.bioinformatics.babraham.ac.uk/projects/trim_galore/), a wrapper tool around Cutadapt [27] and FastQC (https://www.bioinformatics.babraham.ac.uk/projects/fastqc/). Then, the trimmed reads were aligned, per sequencing lane, to the bisulfite-converted GRCh37 reference genome using DRAGEN EP v2.6.3 or later in paired-end mode using the directional/Lister methylation protocol presets; alignments were calculated for both strands, and the unique alignment with highest quality was retained. Lane bam files were merged and then de-duplicated using Picard (version 2.9). A genome-wide cytosine methylation report was generated by DRAGEN to record counts of methylated and unmethylated cytosines at each cytosine position in the genome. Methylation counts are provided for the CpG, CHG and CHH cytosine contexts. DNA methylation level of each CpG was calculated by the number of methylated reads over the total number of sequenced reads. CpGs that were located within sex chromosomes, overlapping with SNPs (dbSNP 137), the DAC Blacklisted Regions or Duke Excluded Regions (generated by the ENCODE project) were removed. CpG sites with less than 20X read coverage were also discarded.

Variants/SNPs (including homozygous alternate and heterozygous genotypes) were inferred using Bis-SNP (version 0.82.2) [28] on the de-duplicated bam files. The homozygous reference genotypes of individuals on these SNPs were extracted from the aligned bam files by requiring >  = 10X read coverage aligned to the reference allele. Hierarchical clustering was performed based on the genotype profiles of SNPs on chromosome 1 where genotypes were inferred from all samples.

### Statistical analysis

A linear regression model (LM) was built to investigate the association between the changes of DNA methylation level before and after treatment (delta beta) and the changes of IGF1 level (delta IGF1) by correcting age onset, the treatment duration and the estimated blood cell proportions. We used the R function lm() to fit the model and calculated *p* values for variables of interest. Due to limited sample size, the nominal *p* value < 1e−4 was used as a threshold of statistical significance to determine differentially methylated CpGs (DMCs). To reduce the impact of zero-inflated methylation differences on inferring the regression, we limited our analysis on CpGs that have >  = 30% of pairs with nonzero methylation differences. Blood deconvolution was done using constrained linear projection [29] via the projectMix function of the RefFreeEWAS package, using a custom panel of 30,455 cell-type-specific hypo-methylated and hyper-methylated CpGs. The blood reference epigenome profiles include neutrophil, monocyte, B cell and T cell.

### Genome features and function enrichment analysis

We downloaded the genome feature annotation tables, including transcription start sites (TSSs), 3’UTRs, 5’UTRs, first exons, exons, introns and transcription end sites (TESs), from the UCSC genome browser with the hg19 build version (https://genome.ucsc.edu/). We considered both TSS200 (200 bp from TSSs) and TSS1500 (1500 bp from TSSs) for the promoter regions. We also downloaded the CpG islands (CGI) annotation table from the UCSC genome browser. Furthermore, CGI north and south shores were defined as the 2-kb flanking sequences on upstream and downstream of CGIs, respectively, and north and south shelves were defined as the 2-kb flanking sequences beyond the shores. Genome feature enrichment analyses of GH response DMCs were performed using Fisher’s exact test for significance where the background set was the all testable CpGs. The gene ontology enrichment analyses were performed using homer [30] (version 4.11) with gene sets detected from the immune panel as the background set.

## Results

### Descriptions of the GH cohort

Main characteristics of the 47 subjects (pairs) included in this study are shown in Table 1. All had ISS as defined by [31]. Briefly, ISS is a condition in which the height of the individual is more than 2 standard deviations (SD) below the corresponding mean height for a given age, sex and population, in whom no identifiable disorder is present.

**Table 1:** Main characteristics of the studied children

	Mean ± SD
*N*	47
Sex (*M*/*F*)	23/24
Age at rhGH onset (year)	9.8 ± 1.8
Tanner stage (stage 0/stage 1)	36/11
Mean rhGH dose (µg/kg day)	70.8 ± 19
Treatment duration (months)	18.1 ± 7.3
Growth rate before treatment (cm/year)	4.6 ± 1.0
Growth rate during treatment (cm/year)	9.1 ± 1.5
Plasma IGF1 at baseline (ng/ml)	167 ± 80
Mean IGF1during treatment (ng/ml)	402 ± 123
Delta IGF1 under treatment (ng/ml)	235 ± 117

The sex-matched participants (24 females vs. 23 males) were aged 4.9 to 13.1 years at time of starting rhGH treatment. They received a mean supra-physiological dose of rhGH of 70.8 μg/kg per day for a duration range from 6 to 38.4 months. During rhGH therapy, the mean increase in plasma IGF1 across children ranged from 4 to 658 ng/ml.

### Differentially methylated CpGs in response to GH treatment

The average sequence genome coverage in targeted regions for these 94 samples was 25-fold (Additional file 1: Table S1). Close to 5.3 million CpGs (including those out of the targeted panels) were captured at autosomes with more than 20-fold read coverage and in at least one sample. When restricting attention to CpGs with good sample coverage in at least 10 before and after treatment pairs, 3,342,494 CpGs at autosomes remained for downstream analysis. In addition, the paired samples before and after GH treatment for 47 children were clustered well according to their genotype profiles which were inferred from the methylation sequencing data (Additional file 2: Figure S1).

**Fig. 1:**
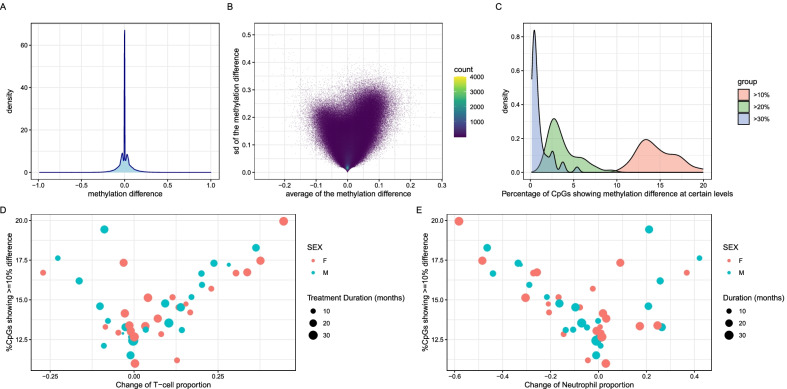
Characterization of DNA methylation changes before and after GH stimulation treatment. **A** The distribution of the DNA methylation changes. **B** The scatter plot between the DNA methylation changes and standard deviation of DNA methylation changes over all CpGs. The density of CpGs was also illustrated using different colors as indicated in the legend. **C** The distribution of the percentage of CpGs showing different level of differential methylation changes (> 10%, > 20% and > 30%) across samples. **D**, **E** The correlation between the percentage of DMCs (at > 10% methylation level difference) and the changes of T cell proportion (**D**) and neutrophil proportion (**E**). The sex and treatment duration were indicated with different colors and different sizes of the dots

We first compared the DNA methylation difference of each CpG per individual pair. For these ~ 3.3 M CpGs across 47 individual pairs, 68.1% of all comparisons were measured, see the sample (pair) coverage distribution of CpGs in Additional file 3: Figure S2A. A majority of the CpGs showed low mean absolute methylation difference (e.g., > 95% of these CpGs are showing mean methylation differences ≤ 5%) and low standard deviation (Fig. 1A, B). Specifically, it showed majority of them have DNA methylation difference of zero as indicated in the middle peak of Fig. 1A. Meanwhile, it also showed two lower sub-peaks which indicated the potential epigenetic responses to the GH treatment and represent the average methylation changes (i.e., ~  ± 5%). When considering each of the measured CpG pairs across 47 children (*n* = 107,019,585), the mean absolute value of the methylation changes between the baseline and rhGH stimulated samples is 4.8 ± 6.6% (SD). There is no difference between male and female groups (Additional file 3: Figure S2B). By requiring ≥ 10% methylation difference between the baseline and rhGH stimulated samples, 304,528 CpGs showed either ≥ 10 hypo-methylated samples or ≥ 10 hyper-methylated samples. Among those CpGs, 37,128 CpGs showed discordant responses to rhGH treatment (i.e., ≥ 10 hypo-methylated samples and ≥ 10 hyper-methylated samples). Furthermore, the corresponded numbers of CpGs drop to 11,398 and 0 if requiring ≥ 20% methylation changes. Overall, we observed quite heterogeneous DNA methylation responses to rhGH treatment. Additional file 3: Figure S2C demonstrates the methylation changes pattern for the top 5% most variable CpGs whose methylation profiles were measured for all individuals.

We observed that roughly 14.64%, 3.76% and 1.07% of the CpGs showed methylation difference > 10%, 20% and 30% comparing the baseline and after treatment samples, respectively (Fig. 1C). We then correlated the ratio of CpGs showing methylation difference > 10% of individuals with other phenotypes such as onsite age, difference of blood proportions and changes in plasma IGF1 concentration under treatment. We observed that the proportion changes of T cell and neutrophil are significantly corrected with the ratio of CpGs showing >  = 10% differences (*r* = 0.39 and − 0.32, with *p* = 0.006 and 0.03, respectively) (Fig. 1D, E), but not for others parameters. This correlation trends are further increased when checking the ratio of CpGs showing >  = 20% or 30% methylation level changes (Additional file 1: Table S2). Furthermore, we also observed that the proportion changes of B cell are showing significant correlation with the ratio CpGs showing 30% methylation level changes. Additional file 1: Table S2 presents the detailed correlations between CpG ratios at different levels of methylation changes and various phenotypic features. This implied that the methylation level changes might be confounded by the blood proportions changes.

**Fig. 2:**
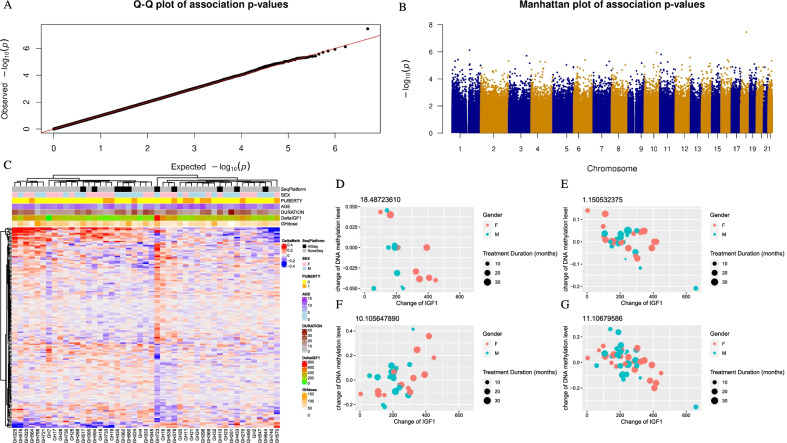
The distribution of response-dependent differentially methylated CpGs. **A** The QQ-plot of *p* values from the analysis of the CpGs respond to the GH treatment. **B** Manhattan plot of *p* values from the response analysis. **C** The heatmap of response DMCs at *p* value < 1e−3 whose methylation profiles were measured for all individuals. Different phenotype features (including different sequencing platforms, sex, puberty, age onset, treatment duration, changes of IGF1 concentration and GH dose) are illustrated in the top plots. **D**–**G** Scatter plot for the examples of top response DMCs. The sex and treatment duration were indicated with different colors and different sizes of the dots

To identify the differentially methylated CpGs upon long-term exposure to rhGH, we fit a linear regression model on the difference between the DNA methylation values of all baseline and post-treatment PBMC samples and the difference in IGF1 values (Delta-IGF1) by adjusting the age at onset of treatment, treatment duration and differences in blood cell proportions before and after treatment. Figure 2A, B illustrates the QQ-plot and the Manhattan plot for this analysis. We did not observe any significant associations between Delta-IGF1 and the methylation level changes of CpGs (response DMCs) between baseline and post-treatment samples after multiple test corrections at a FDR of 0.05. However, we identified 2599 CpGs showing response DMCs (*p* < 1e−3) among baseline and post-treatment samples where 1317 response DMCs are negatively correlated and 1282 DMCs are positively correlated. With a more stringent *p* value threshold, we identified 267 DMCs at *p* value < 1e−4 with 123 negatively correlated and 144 positively correlated response DMCs. The DNA methylation change’s pattern of the DMCs at *p* < 1e−3, which were also measured by all the individuals, is illustrated in Fig. 2C. Table 2 lists the top 20 significant response DMCs with the top examples demonstrated in Fig. 2D–G, and Additional file 1: Table S3 lists the full list of these 267 response DMCs.

**Table 2:** The top 20 response DMCs list with *p* value < 1e−4. The response DMCs were sorted by the *p* value. CpG chromosome and position, regression *p* value, beta value (coefficient) and the annotated closest gene information (including genomic Annotation, Distance to TSS, Gene Name, Gene Type, and Gene Description of the closest gene) were provided

chr.position	*p* value	Beta	Annotation	Distance to TSS	*Gene Name*	Gene type	Gene description
chr18.48723610	3.49E−08	− 0.22733	exon (NM_016626, exon 1 of 2)	440	*MEX3C*	Protein-coding	mex-3 RNA binding family member C
chr1.150532375	7.49E−07	− 0.39527	TTS (NR_104133)	7971	*MIR4257*	ncRNA	microRNA 4257
chr10.105647890	1.17E−06	0.893509	intron (NM_024928, intron 9 of 9)	30,051	*STN1*	Protein-coding	STN1 subunit of CST complex
chr11.10679586	1.56E−06	− 0.58556	intron (NM_001206880, intron 1 of 19)	− 5739	*MRVI1*	Protein-coding	Murine retrovirus integration site 1 homolog
chr3.156848563	1.97E−06	− 0.14999	Intergenic	− 7773	*LINC00880*	ncRNA	Long intergenic non-protein-coding RNA 880
chr12.129252225	2.68E−06	− 1.11703	Intergenic	56,277	*SLC15A4*	Protein-coding	Solute carrier family 15 member 4
chr1.50834156	3.76E−06	0.153745	Intergenic	54,958	*DMRTA2*	Protein-coding	DMRT-like family A2
chr16.56553641	3.90E−06	− 0.13719	intron (NM_031885, intron 1 of 16)	294	*BBS2*	Protein-coding	Bardet-Biedl syndrome 2
chr6.99283220	3.95E−06	− 0.12451	exon (NM_005604, exon 1 of 1)	771	*POU3F2*	Protein-coding	POU class 3 homeobox 2
chr22.46519089	4.61E−06	− 1.1146	Intergenic	9524	*MIRLET7B*	ncRNA	microRNA let-7b
chr1.25228801	4.78E−06	− 0.30407	exon (NM_004350, exon 5 of 5)	17,105	*MIR6731*	ncRNA	microRNA 6731
chr2.241976241	4.88E−06	− 0.48453	exon (NM_001080437, exon 5 of 32)	38,207	*SNED1*	Protein-coding	Sushi, nidogen and EGF-like domains 1
chr7.139187227	5.05E−06	− 0.39751	Intergenic	− 18,809	*KLRG2*	Protein-coding	Killer cell lectin-like receptor G2
chr4.79545349	5.32E−06	0.366908	Intergenic	− 21,798	*LINC01094*	ncRNA	Long intergenic non-protein-coding RNA 1094
chr16.66554996	5.35E−06	0.684785	intron (NR_073520, intron 5 of 8)	29,028	*TK2*	Protein-coding	Thymidine kinase 2
chr11.68608981	5.63E−06	− 0.13725	intron (NM_001876, intron 1 of 18)	402	*CPT1A*	Protein-coding	Carnitine palmitoyltransferase 1A
chr4.122871430	5.71E−06	0.274039	intron (NM_001366479, intron 1 of 10)	1784	*TRPC3*	Protein-coding	Transient receptor potential cation channel subfamily C member 3
chr11.8364991	6.74E−06	0.562738	Intergenic	− 74,658	*LMO1*	Protein-coding	LIM domain only 1
chr9.117026652	6.75E−06	1.475667	exon (NM_032888, exon 29 of 61)	54,939	*MIR455*	ncRNA	microRNA 455
chr3.183873096	6.93E−06	0.345301	promoter-TSS (NM_004423)	− 68	*DVL3*	Protein-coding	Disheveled segment polarity protein 3

### Genome feature and function enrichment analysis of response DMCs

**Fig. 3:**
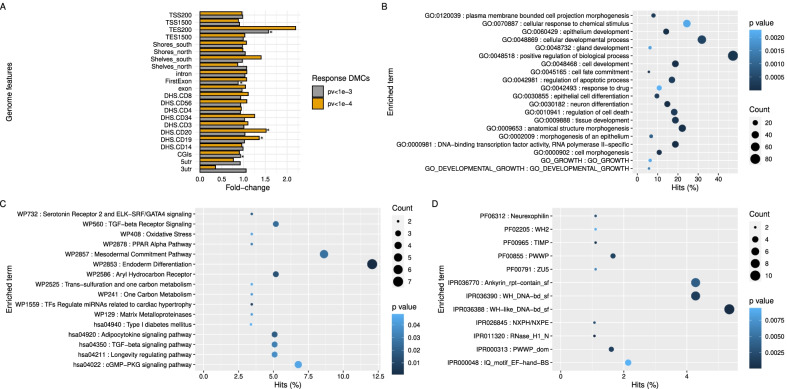
Genome feature and functional enrichment analysis of the response DMCs. **A** Genomic features and blood regulatory element enrichment analysis of the response DMCs with *p* value < 1e−3 and *p* value < 1e−4. Fisher test: *: *p* value < 0.01. **B**–**D** Functional enrichment analysis of the response DMCs. Enrichment of functional grouping of genes through the biological process, groups of the genes in the same pathway through KEGG, pathway interaction database as well as the WikiPathways, and the similar domain and features of the gene’s product proteins through PFAM and Interpro domain database were illustrated in (**B**), (**C**) and (**D**), respectively. The number of genes in each item and *p* value of the enrichment analysis was shown in the legend

We first performed a genomic feature enrichment analysis of response DMCs with the *p* value < 1e−4 and *p* value < 1e−3. We observed that the response DMCs with *p* value < 1e−3 were slightly enriched in the first exon, TES200 and CGIs (Fig. 3A) but no significant regions for the response DMCs with *p* value < 1e−4 (Fig. 3A). Furthermore, we also observed slightly enrichments on the blood-specific regulatory elements—DNase I hypersensitive sites (DHSs) regions of CD19 and CD20 for the response DMCs with *p* value < 1e−4 but not for the response DMCs with *p* value < 1e−3 (Fig. 3A).

We then performed a gene function enrichment analysis on 265 genes which are associated with 267 *p* value < 1e−4 response DMCs using Homer (annotatepeaks function) [30]. It revealed that these genes were enriched in the Biological process GO term of “neuron differentiation” (*p* value < 1e−05), “cell development” and “cell morphogenesis” (*p* value < 1e−4), as well as other developmental process-related terms, including the “Development growth” and “Growth” terms (*p* value < 3e−3) **(**Fig. 3B). Strikingly, these genes were observed to be enriched in the “Endoderm differentiation pathway” (*p* value < 1e−3), “Adipocytokine signaling pathway” (*p* value = 0.019), “TGF-beta signaling pathway” (*p* value = 0.02) and “PPAR Alpha pathway” (*p* value < 0.03) as well as other pathways related to growth factor receptor or stimulating hormone signaling pathway **(**Fig. 3C). Furthermore, the common domain families of “Rnase H1,” “WH/WH-like DNA” (*p* value < 1e−4), “TIMP,” “PWWP,” etc. (*p* value < 1e−3), were observed to be over-represented in the proteins of these response DMCs-associated genes (Fig. 3D). In addition, when exploring the enrichment for genes associated with response DMCs at *p* < 1e−3 (*n* = 2247 genes), we observed that these genes were enriched in “PI3K-Akt signaling pathway,” “Sphingolipid signaling pathway” and “cAMP signaling pathway” (Additional file 4: Figure S3).

We compared our identified DMCs with previously reported GH-related DMCs (*n* = 239, [32]). We did not find that any reported significant DMCs were replicated at our study, but when expanded to 1 kb distance-based neighboring CpGs, we observed a slight enrichment (fold-change of 1.73) of these reported DMCs at our response DMCs (*p* value < 0.001) compared with our non-significant CpGs (*p* value > 0.001) although this enrichment was not significant (*p* value = 0.15) due to the small number of overlapped CpGs being counted. Interestingly, one response DMC (with *p* value = 1e−5) at chr11:68608981, which is located within the *CTP1A* gene, was within 200 bp to the reported GH DMC at chr11:68609166 (*p* value = 1e−5, [32]).

It was reported that the effect of GH treatment on growth is potentially influenced by individual’s SNPs [33]. We first linked our response DMCs to 37 potential SNPs which were reported to be GH-response-associated SNPs. We observed that only 14 SNPs have their surrounding CpGs in our tested CpG sets within 5 kb distance. Interestingly, three of these 14 potential SNPs were showing significant response DMCs (*p* value < 0.01) within a 5 kb distance to these SNPs. Particularly, one SNP (rs6600230, chr16:738477) is overlapping with the gene *WDR24* where multiple CpGs were showing *p* value < 0.05 with the significant one (chr16:739598) located at the first exon region (*p* value = 0.01).

## Discussion

The emerging field of pharmacoepigenomics will provide promising insights into the role drugs play in modulating the host epigenome and in addressing inter-individual variability in drug response and adverse effects. Although there is growing evidence that pharmacoepigenetics has the potential to become an important element of personalized medicine, we know of no study that has evaluated the changes in the individual methylome of the same group of patients undergoing a treatment, as performed in the current study. An additional advantage of our pharmacoepigenetic study is that clinical (growth rate, height) and biological outcomes (IGF1) can be quantified in response to precise rhGH dosing carefully injected by parents. More specifically, our data provide the first comprehensive pharmacoepigenomics analysis on rhGH treatment in children with ISS by comparing DNA methylation marks before and after several months of rhGH treatment. We identified 267 response DMCs which are associated with 265 genes and these genes were enriched in the biological process of cell differentiation, system development and different growth-related pathways such as endoderm differentiation, adipocytokine signaling, PPAR alpha and TGF-beta signaling pathways. This pilot study thus supports the existence of dynamic epigenetic changes in response to rhGH treatment. Again, it should be recalled that these are methylation changes induced by prolonged GH administration, and not epigenetic marks associated with short stature, an example of which can be found in one of our previous studies [34].

Our customized methylation sequencing panel captured more than 5 million CpGs, which is much larger than the previously used 450 K array data (i.e., > 10 folder larger), representing an unprecedented level of resolution. After quality control, more than 3.3 million CpGs remained for the response association analysis, providing the potential to discover novel signals. Indeed, of identified 267 response DMCs (with *p* value < 1e−4), only 114 (43%) of them are located within 100 bp distance to the known 450 K loci and only 25 (9.4%) of them are exactly located at the 450 K loci. Among the top 20 response DMCs listed in Table 2, half of them does not have neighboring CpGs (with 100 bp distance) in 450 K loci.

Previous studies had shown that the *TGF*-beta signaling pathway plays an important role in regulating osteoblast differentiation and could inhabit *IGF*-1/*Akt* signaling pathway [35]. The adipocytokine pathway and *cAMP*-signaling pathway are downstream signaling pathways upon activation of *IGF1* receptor and contribute to the signal transduction of insulin-like growth factors on growth [36]. Another study reported that the GH modulates *EGFR* expression and signaling and further activates *PI3K*-*Akt* signaling, which was enriched in our response DMCs (*p* value < 1e−3) [37]. Moreover, our response DMC-associated genes were enriched in DNA-binding transcription factors as well as proteins with the common domain families of “WH/WH-like DNA” and “TIMP.” Particularly, TIMP3 is known to modulate GHR abundance and GH sensitivity [38], and *NFKB1* is a known gene associated with short stature [39] and the growth-promoting effects of the transcription factor family of *NFKB* seems to be facilitated by GH and IGF-1[40], while *FOXA1*, *FOXN1* are regulators for GH activation [41]. Here, our identified genes with rhGH-associated methylation changes were enriched in these pathways, supporting the biological relevance of our findings. The genes involved in these pathways include *CDKN2B, LEFTY2, PPP2R1B, CPT1A, RXRA, NFKB1, KCNMA1, BORCS8-MEF2B, MRVI1, PPIF and GATA4* (See the full response DMC list at *p* value < 1e−3, Additional file 1: Table S4).

The current study identified marked intra-individual responses of DNA-methylation to long-term rhGH treatment. A study by Kolarova et al. investigated 24 patients at baseline and after only 4 days of rhGH administration [32]. The studied patients had various forms of GH deficiency (*N* = 13) or other pathological conditions, which could influence the epigenetic responses to rhGH and complicate the interpretation. In comparison, only healthy children were selected for the current study and were either prepubertal or with minimal manifestations of puberty in order to avoid epigenetic changes that are associated in blood cells with advancing puberty [42].

Array-based DNA-methylation profiling of paired peripheral blood mononuclear cell samples in the Kolarova et al.’s study revealed clustering according to individuals rather than treatment [32]. Supervised analysis identified 239 CpGs as significantly differentially methylated between baseline and acutely GH-stimulated samples, which nevertheless did not retain significance after adjustment for multivariate analysis. In a companion study, Kolarova et al. investigated the long-term effects of prolonged rhGH treatment on the DNA-methylome and analyzed peripheral blood cells from an independent cohort of 36 rhGH-treated children born small for gestational age (SGA) compared to 18 untreated controls. These were not paired samples which had to face major unwanted inter-individual variance of children methylome. No differentially methylated targets reached the level of significance in this long-term rhGH-treated cohort [32]. Our study did not replicate any of these 239 DMCs but observed a slight enrichment if considering significant response DMCs in 1 kb distance to them. The lack of high replicates might due to different etiologies of short stature (intrauterine growth retardation may influence epigenetic marks in the Kolarova et al.’s study) design, different treatment durations and dosing, different ages and more importantly the considerable inter-individual heterogeneity, while our study investigated paired intra-individual changes in methylation.

Of interest, *MEX3C*, the top gene in our response association analysis, was reported to be a translational regulator of *IGF* expression in mice [43]. IGF1 protein expression in bone cells was decreased upon *MEX3C* deficiency in *Mex3c* homozygous mutant mice. Given that *MEX3C* is highly conserved among mammalian species, the observation in mice might be relevant to the human IGF1 regulation and warrants further investigation. Among the top 20 signals, the response DMC at *CPT1A* (chr11:68608981) was close to the reported locus (cg20228509, chr11:68609166) within 1 kb in the Kolarova et al.’s short-term rhGH treatment study [32]. *CPT1A* was observed to be a genetic regulation of fatty acid metabolism, and missense mutation reduces height [44]. Although this evidence was not revealed in epigenetic studies, the potential pathway *CPT1A* involved (such as the adipocytokine signaling pathway, an important pathway related to IGF signaling) might indicate its indirect association with GH. In the same study, more than 3 CpGs in *SLC15A4* were identified as differentially methylated loci and two of them were further validated with bisulfite pyrosequencing [32]. Our data showed the top signal was at the downstream of *SLC15A4*, and a few CpGs with nominal significance at *p* value < 0.01 were located in the intron of *SLC15A4* gene. In addition, we also observed a couple of non-coding RNAs at our top signals list. Their functions related to GH are currently unknown and need further exploration.

As in almost all epigenetic studies in humans, we were only able to characterize DNA methylation in blood cells, which may not recapitulate all GH-induced changes that may occur in other target cells. However, PBMC are sensitive to the GH/IGF1 axis [45] and may thus reveal epigenetic changes triggered by GH or IGF1. B-lymphocytes and monocytes as well as T-lymphocytes and natural killer cells express GH receptors on their cell surfaces [46, 47]. These cells also express IGF1 receptors [48], which activate the mTOR pathway and can subsequently induce epigenomic changes [49]. GH [50–52] and IGF1 signaling [53, 54] have been studied in PBMC and lymphocytes. Since the top variable CpGs in our study were highly associated with the proportion changes of T cell and neutrophils, we applied a well-established computational approach to deconvolute the PBMC blood compositions and included them as covariates in our analysis model, which would effectively remove the confounders due to dynamic blood cell proportion changes. Finally, the current study supports the utility of PBMC to detect DNA methylation changes responding to rhGH treatment.

In summary, we have identified multiple response DMCs that are associated with rhGH treatment although none of them show the FDR significance after multiple testing correction. This is most likely due to the limited sample size given the large inter-individual variation in DNA methylation changes, which restricted our power to detect significant associations at FDR q-value threshold of 0.05. The downstream functional analysis revealed that the response DMCs were enriched in many pathways biologically relevant to GH. Larger sample sizes will be needed to more definitively identify epigenetic changes arising from rhGH administration. Further functional genomics investigations are also encouraged for validation of our discoveries, particularly for the top signals.

## Supplementary Information

**Additional file 1: Table S1**. Sequence statistics for the GH samples. **Table S2**. Correlations between DMC ratios and other meta data features. The ratio of CpGs showing >  = 10%, >  = 20%, >  = 30% methylation level changes against other features. **Table S3.** Full list of response DMCs at *p* value < 1e−4. The chromosome and position of CpGs, regression *p* value, beta value (coefficient), and the annotated closest gene information (including genomic Annotation, Distance to TSS, Gene Name, Gene Type, and Gene Description of the closest gene) were provided. **Table S4**. Full list of response DMCs at *p* value < 1e−3. The chromosome and position of CpGs, regression *p* value, beta value (coefficient), and the annotated closest gene information (including genomic Annotation, Distance to TSS, Gene Name, Gene Type, and Gene Description of the closest gene) were provided.

**Additional file 2: Figure S1**. Hierarchical clustering of 94 samples based on their genotype profiles. Genotype profiles of SNPs measured for all the 94 samples were used to perform the hierarchical clustering. Manhattan distance was used to calculate the distance between two samples. T0: baseline; T1: second time point (treatment).

**Additional file 3: Figure S2**. Characterization of DNA methylation changes. **(A)** Individual coverage of CpGs which have both before and after treatment measures in this cohort. **(B)** The distribution of the DNA methylation changes per sex. Male and female were indicated in the legend. **(C)** The heatmap of top 5% variable CpGs whose methylation profiles were measured for all individuals. Different phenotype features (including different sequencing platforms, sex, puberty, age onset, treatment duration, changes of IGF1 concentration and GH dose) are illustrated in the top plots.

**Additional file 4: Figure S3**. Functional enrichment analysis of the response DMCs at *p* value < 1e−3. **(A-C)** Functional enrichment analysis of the response DMCs at *p* value < 1e−3. Enrichment of functional grouping of genes through the biological process, groups of the genes in the same pathway through KEGG, pathway interaction database as well as the WikiPathways, and the similar domain and features of the gene’s product proteins through PFAM and Interpro domain database were illustrated in **(A), (B)** and **(C),** respectively. The number of genes in each item and *p* value of the enrichment analysis were shown in the legend.

## Data Availability

All the raw read files were submitted to European Genome-phenome Archive under the accession number EGAS00001006115.

## Abbreviations

cAMP: Cyclic adenosine 3′, 5′-monophosphate
CGIs: CpG islands
DHS: DNase I hypersensitive sites
DMCs: Differentially methylated CpG sites
ENCODE: Encyclopedia of DNA elements
FDR: False discovery rate
GH: Growth hormone
GO: Gene ontology
IGF1: Insulin-like growth factor 1
ISS: Idiopathic short stature
LM: Linear regression
MCC-Seq: MethylC-capture sequencing
PBMC: Peripheral blood mononuclear cells
PDGF: Platelet-derived growth factor
PKC: Protein kinase C
rhGH: Recombinant human growth hormone
SD: Standard deviation
SGA: Small for gestational age
T1D: Type 1 diabetes
TGF-beta: Transforming growth factor beta
TIMP: Tissue inhibitors of matrix metalloproteinase
TSS: Transcription start site
