# Supplementary figures and images for: Differentially methylated CpGs in response to growth hormone administration in children with idiopathic short stature
Source: Clin Epigenetics. 2022 May 18;14:65. doi: 10.1186/s13148-022-01281-z (PMC9118695; doi:10.1186/s13148-022-01281-z)

Cluster Dendrogram

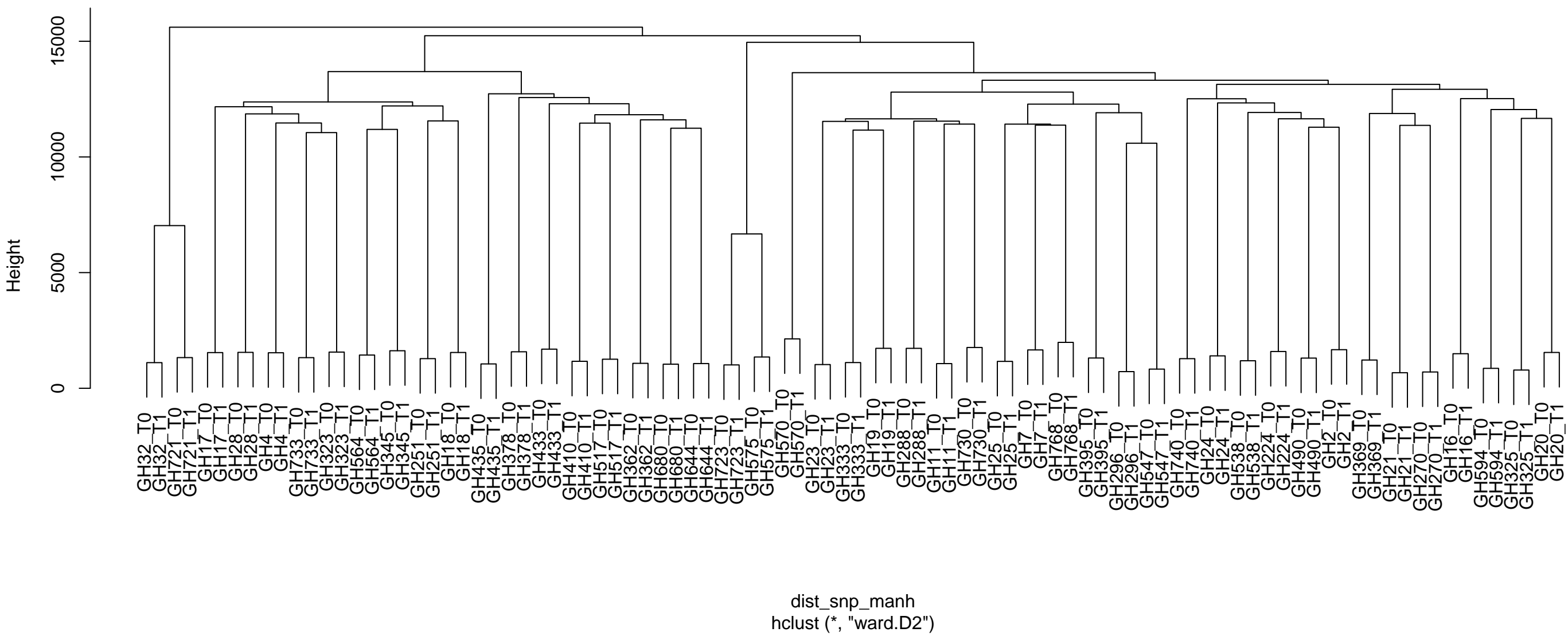

Supplement: Supplementary file 2 — Additional file 2: Figure S1. Hierarchical clustering of 94 samples based on their genotype profiles. Genotype profiles of SNPs measured for all the 94 samples were used to perform the hierarchical clustering. Manhattan distance was used to calculate the distance between two samples. T0: baseline; T1: second time point (treatment). [file 13148_2022_1281_MOESM2_ESM.pdf]

A

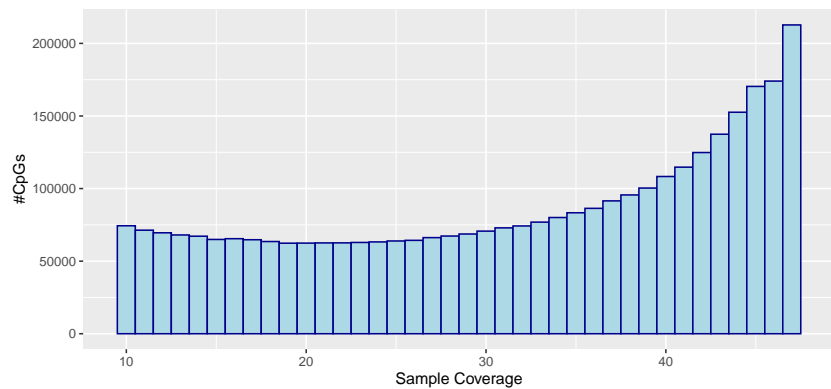

B

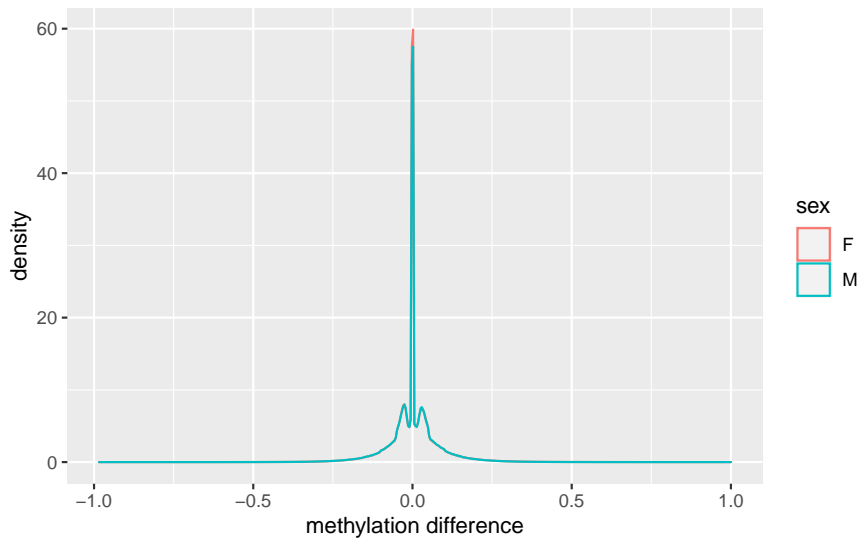

C

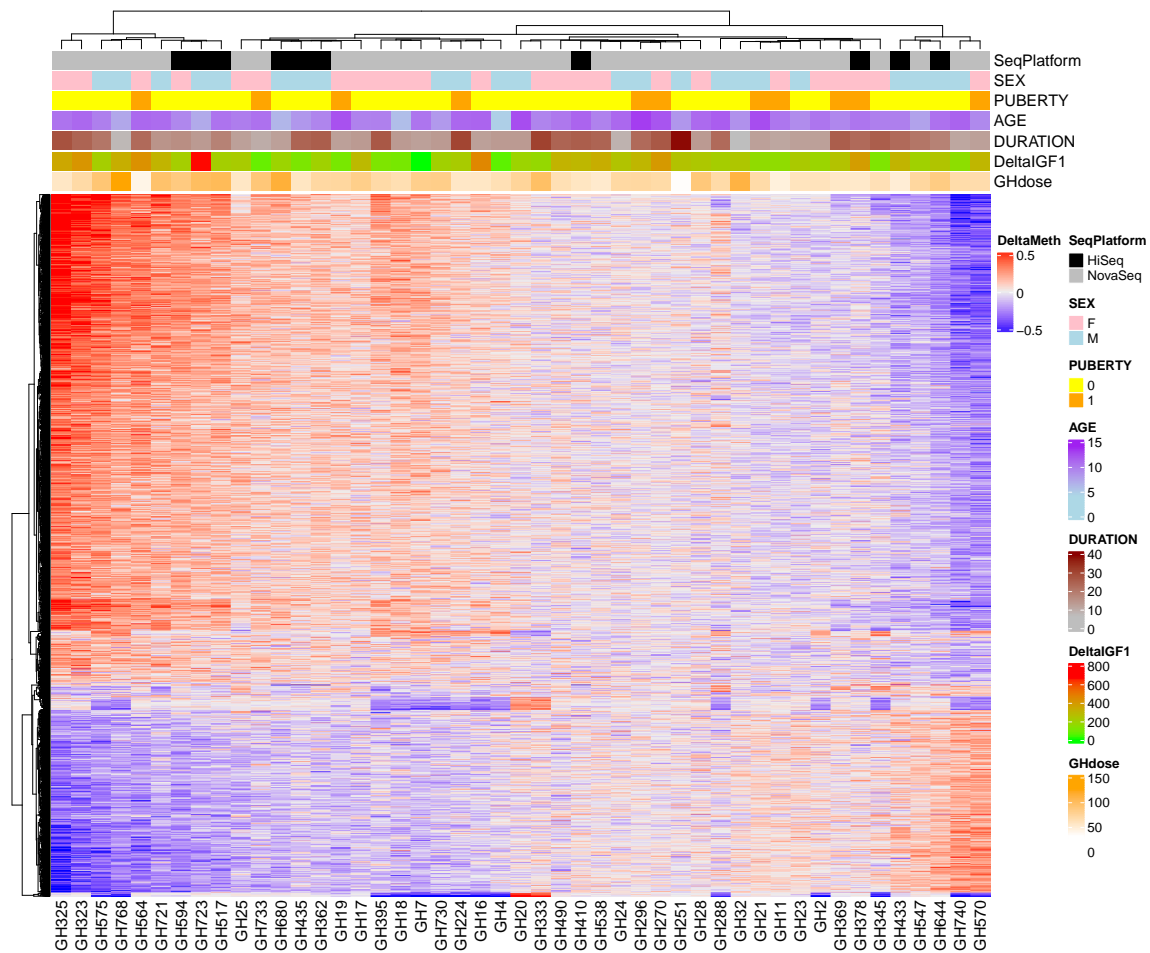

Supplement: Supplementary file 3 — Additional file 3: Figure S2. Characterization of DNA methylation changes. (A) Individual coverage of CpGs which have both before and after treatment measures in this cohort. (B) The distribution of the DNA methylation changes per sex. Male and female were indicated in the legend. (C) The heatmap of top 5% variable CpGs whose methylation profiles were measured for all individuals. Different phenotype features (including different sequencing platforms, sex, puberty, age onset, treatment duration, changes of IGF1 concentration and GH dose) are illustrated in the top plots. [file 13148_2022_1281_MOESM3_ESM.pdf]

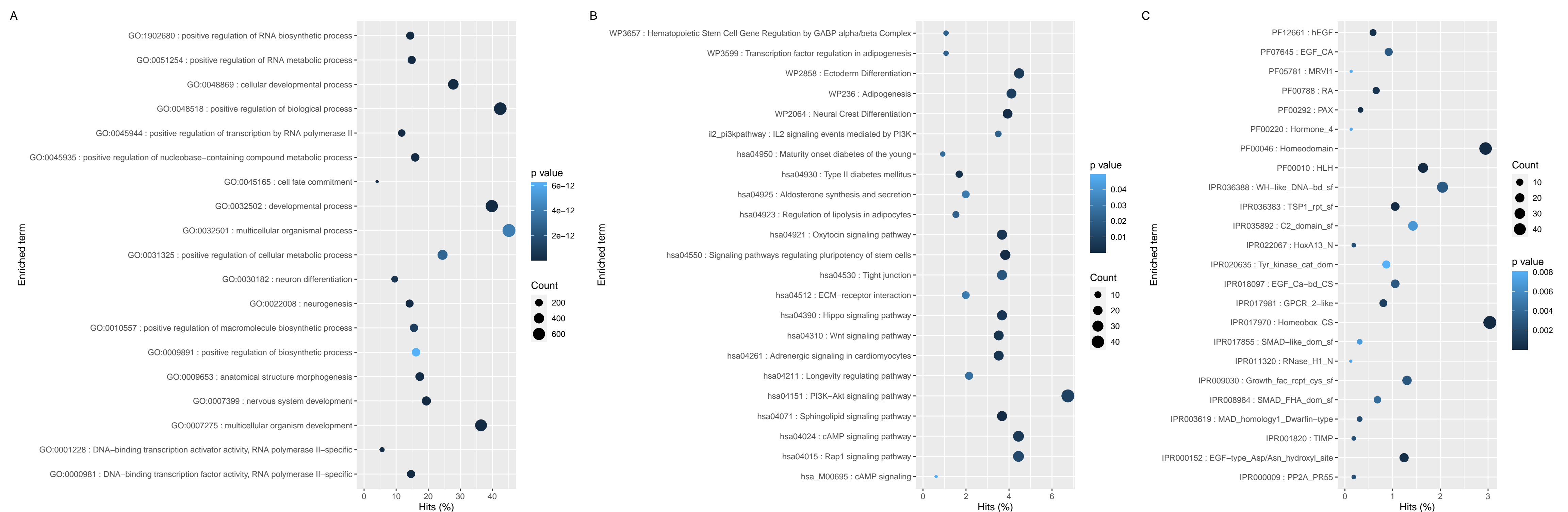

Supplement: Supplementary file 4 — Additional file 4: Figure S3. Functional enrichment analysis of the response DMCs at p value < 1e−3. (A-C) Functional enrichment analysis of the response DMCs at p value < 1e−3. Enrichment of functional grouping of genes through the biological process, groups of the genes in the same pathway through KEGG, pathway interaction database as well as the WikiPathways, and the similar domain and features of the gene’s product proteins through PFAM and Interpro domain database were illustrated in (A), (B) and (C), respectively. The number of genes in each item and p value of the enrichment analysis were shown in the legend. [file 13148_2022_1281_MOESM4_ESM.pdf]
